# The Influence of Perioperative Antibiotic Prophylaxis on Wound Infection and on the Colonization of Wound Drains in Patients After Correction of Craniosynostosis

**DOI:** 10.3389/fped.2021.720074

**Published:** 2021-08-24

**Authors:** Johannes Holle, Tobias Finger, Julia Lugonja, Florian Schmidt, Andreas Schaumann, Alexander Gratopp, Ulrich-Wilhelm Thomale, Horst von Bernuth, Matthias Schulz

**Affiliations:** ^1^Department of Pediatric Gastroenterology, Nephrology and Metabolic Diseases, Charité—Universitätsmedizin Berlin, Berlin, Germany; ^2^Pediatric Neurosurgery, Charité—Universitätsmedizin Berlin, Berlin, Germany; ^3^Department of Pediatric Pulmonology, Immunology and Intensive Care Medicine, Charité—Universitätsmedizin Berlin, Berlin, Germany; ^4^Berlin Institute of Health Center for Regenerative Therapies (BCRT), Charité—Universitätsmedizin Berlin, Berlin, Germany; ^5^Department of Immunology, Labor Berlin GmbH, Berlin, Germany

**Keywords:** perioperative antibiotic prophylaxis, wound infection, craniosynostoses/surgery, antibiotic stewardship, drain colonization

## Abstract

**Objective:** Evidence for the duration of perioperative antibiotic prophylaxis (PAP) after the correction of craniosynostosis in children is scarce. We evaluated the necessary duration of PAP to ensure a minimal rate of postoperative wound infections.

**Methods:** In this monocentric, retrospective, and prospective pilot study, two PAP protocols were compared. From August 2017 to May 2018, treatment group 1 (TG 1) was treated using the standard PAP protocol with at least three doses of antibiotics. Between May 2018 and March 2019, a shortened PAP with a single-shot administration was given to treatment group 2 (TG 2a and b). Endpoints of this study were wound infection rate, colonization rate of wound drains, and the course of treatment reflected by clinical and laboratory data.

**Results:** A cohort of 187 children underwent craniosynostosis correction: 167 were treated according to protocols-−95 patients with at least three doses (TG 1) and 72 patients with a single-shot of cefuroxime (TG 2a). Baseline characteristics were similar for both groups. We could not detect significant differences, neither for wound infection rates (TG 1: 1.1%, TG 2a: 0.0%, *p* = 0.38) nor for colonization rates of wound drains (TG 1: 4.8%, TG 2a: 10.5%, *p* = 0.27).

**Conclusions:** Single-shot PAP had no adverse effects on the wound infection rate or the colonization rate of the wound drains compared with prolonged perioperative antibiotic prophylaxis. As a result, single-shot preoperative PAP is now applied to the majority craniosynostosis patients undergoing surgical correction in our unit.

## Introduction

Craniosynostosis, defined as premature fusion of one or more cranial sutures, is rare in children with an estimated prevalence of 3 to 7.2 per 10,000 live births (1–4). The indication for a surgical intervention is based on the extent of the associated phenotype. Because surgical correction is routinely performed in infancy, an early diagnosis as well as a tailored treatment approach, are of paramount importance. To avoid peri- and postoperative complications, especially wound infections and impaired wound healing, perioperative antibiotic prophylaxis is routinely administered to these children.

To date, recommendations for perioperative antibiotic prophylaxis (PAP) for children undergoing craniosynostosis correction can be based mainly on retrospective surveys and cohort studies (5). In the context of antibiotic stewardship and to avoid an inappropriate use of antibiotics, prospective clinical trials are necessary to allow PAP recommendation based on solid evidence.

The presented study aims to provide prospectively collected data about the safety of a reduced perioperative prophylaxis in children upon craniosynostosis surgery. We hypothesize that the reduction of PAP from a 48-h perioperative regimen to a single intraoperative dose does not lead to more frequent postoperative wound infections rate or bacterial colonization of surgical drains.

## Materials and Methods

### Study Design and Population

This combined retrospective-prospective observational study was conducted in a tertiary healthcare center, providing supraregional care for children with craniosynostosis. The study was approved by the local ethics committee (No. EA2/029/18). Written informed consent was obtained from all prospectively enrolled patients and/or their parents.

All surgical procedures were performed under sterile conditions in the operating room with the child under general anesthesia. Postoperatively, the children stayed on a specialized pediatric intensive care unit (PICU) for 24–48 h. All children—aged between 1 month and 18 years—undergoing craniosynostosis correction between August 2017 and March 2019 were included in this study. The minimum clinical follow-up for inclusion into this study was 3 months. Exclusion criteria were immunosuppressive medication, underlying conditions, which might affect immune response or wound healing and a documented intolerance to cefuroxime, which was the routine antibiotic agent.

All children operated on between August 2017 and May 2018 and treated with antibiotic protocol 1 were evaluated retrospectively; whereas the data of children operated on between May 2018 and March 2019 and treated with antibiotic protocol 2 were prospectively collected.

### Data Acquisition

The following variables were evaluated: age, gender, body weight, height, length of hospital stay, duration on the intensive care unit, duration of the operation, diagnosis, selected surgical procedure, intraoperative complications, body temperature, details of antibiotic treatment, results of the microbiological analyses, transfusion and catecholamine requirements, occurrence of wound infection, and duration of follow-up.

Routine preoperative analysis of laboratory parameters included leucocyte count and C-reactive protein (CRP). Postoperatively, procalcitonin (PCT) was additionally analyzed.

### Antibiotic Treatment

PAP was administered using two standardized protocols. Between August 2017 and May 2018, all children were treated according to protocol 1 [treatment group 1 (TG 1)]. These children received an intravenous antibiotic single shot with cefuroxime (50 mg/kg body weight) prior to skin incision. If the time of surgery exceeded 4 h, a second dose was given. Postoperatively, all children treated with protocol 1 received repeated antibiotic prophylaxis with cefuroxime every 8 h (33 mg/kg body weight) until the removal of the surgical site drain at the second postoperative day or, in the absence of a drain, at least for a total of 24 h. Children prospectively enrolled between June 2018 and March 2019 were treated according to protocol 2 (treatment group 2a and b) with a single-shot antibiotic dosage consisting of cefuroxime (50 mg/kg body weight) prior to skin incision. Those children did not receive any further antibiotic prophylaxis postoperatively.

### Microbiological Assessment

Surgical site drains were removed 24–48 h after surgery, depending on the drainage volume. A swab from the surrounding skin was collected before local disinfection. The drain was removed in a sterile fashion, and the tip of the drain was sent for further microbiological analyses. Routine microbiological tests were supplemented by sonication analysis. The removed tip of the drain was transported to the microbiological laboratory in a sterile air-tight container. Sonication was performed within 6 h of removal. After addition of 5 ml normal saline covering the specimen, the container was vortexed for 30 s, sonicated for 1 min at 40 kHz (BactoSonic, Bandelin Electronic, Germany), and vortexed for another 30 s. The resulting sonication fluid was inocculated in a pediatric blood culture bottle. All cultures were incubated at 37 ± 1°C until documented bacterial growth at least for 7 days; anaerobic cultures for at least 14 days.

### Statistical Analysis

All statistical analyses were performed with the SPSS^®^ IBM Corporation (version 25). Normal distribution was assessed by interpretation of histograms, of z-values for skewness and kurtosis, and the Shapiro-Wilk normality test. In the descriptive presentation, normally distributed (parametric) values are presented as mean value (MW) and standard deviation (SD), whereas non-normally distributed values are given as median (MD) and interquartile range (IQR from the 25th to 75th percentiles). The statistical evaluation of qualitative characteristics included the calculation of absolute and percentage values of all valid cases in cross tabulations. For the analysis of differences between the groups, the significance was then checked using the Chi-square test according to Pearson's correlations. A logistic regression analysis was performed to test whether independent variables such as age and duration of surgery have had an influence on the sonication findings. A level of *p* < 0.05 was considered to indicate statistical significance.

## Results

### Study Cohort and Baseline Characteristics

**Figure 1:**
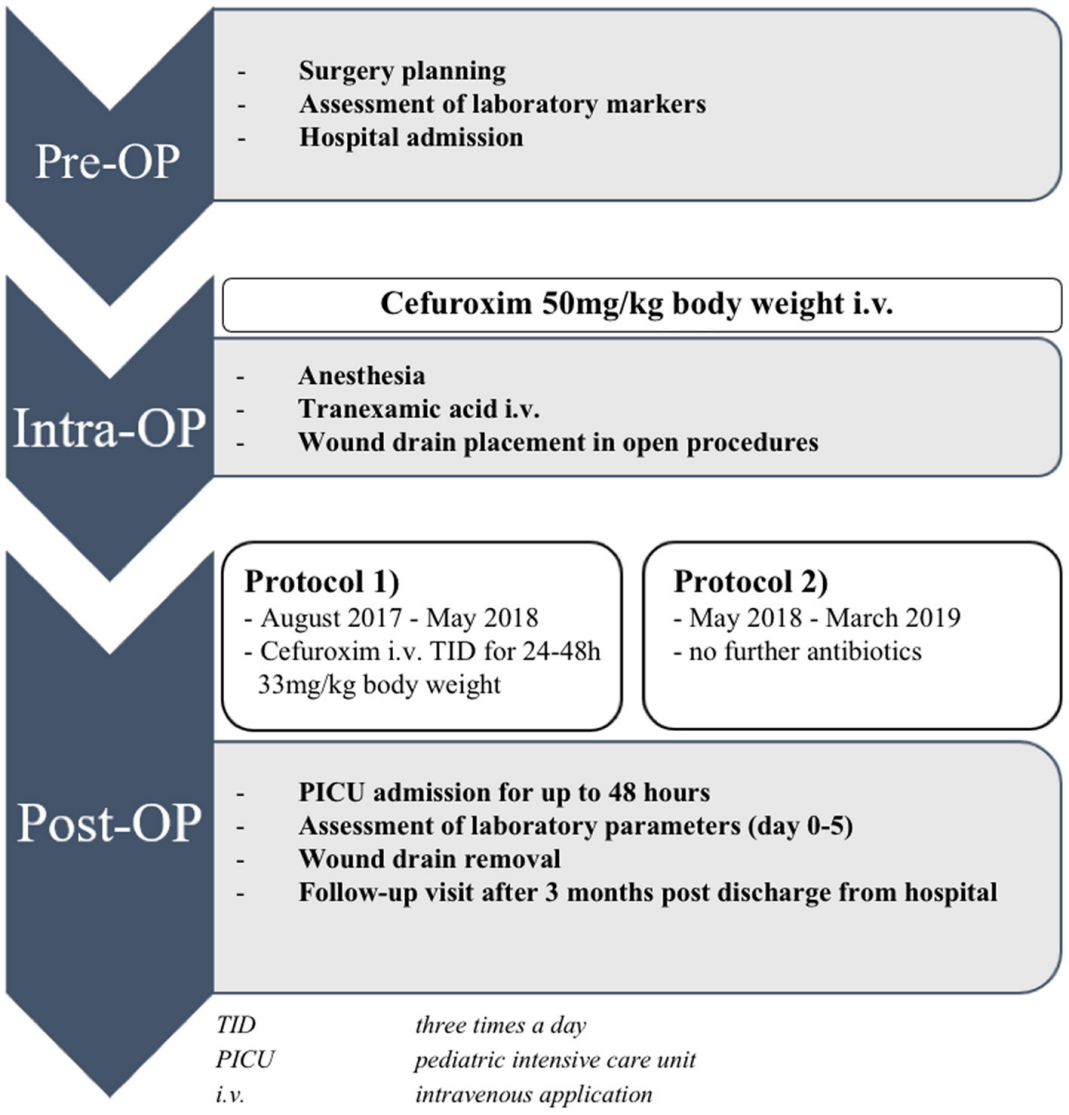
Perioperative procedure of children undergoing elective craniosynostosis correction. TID, three times a day; ICU, intensive care unit; i.v., intravenous application.

**Figure 2:**
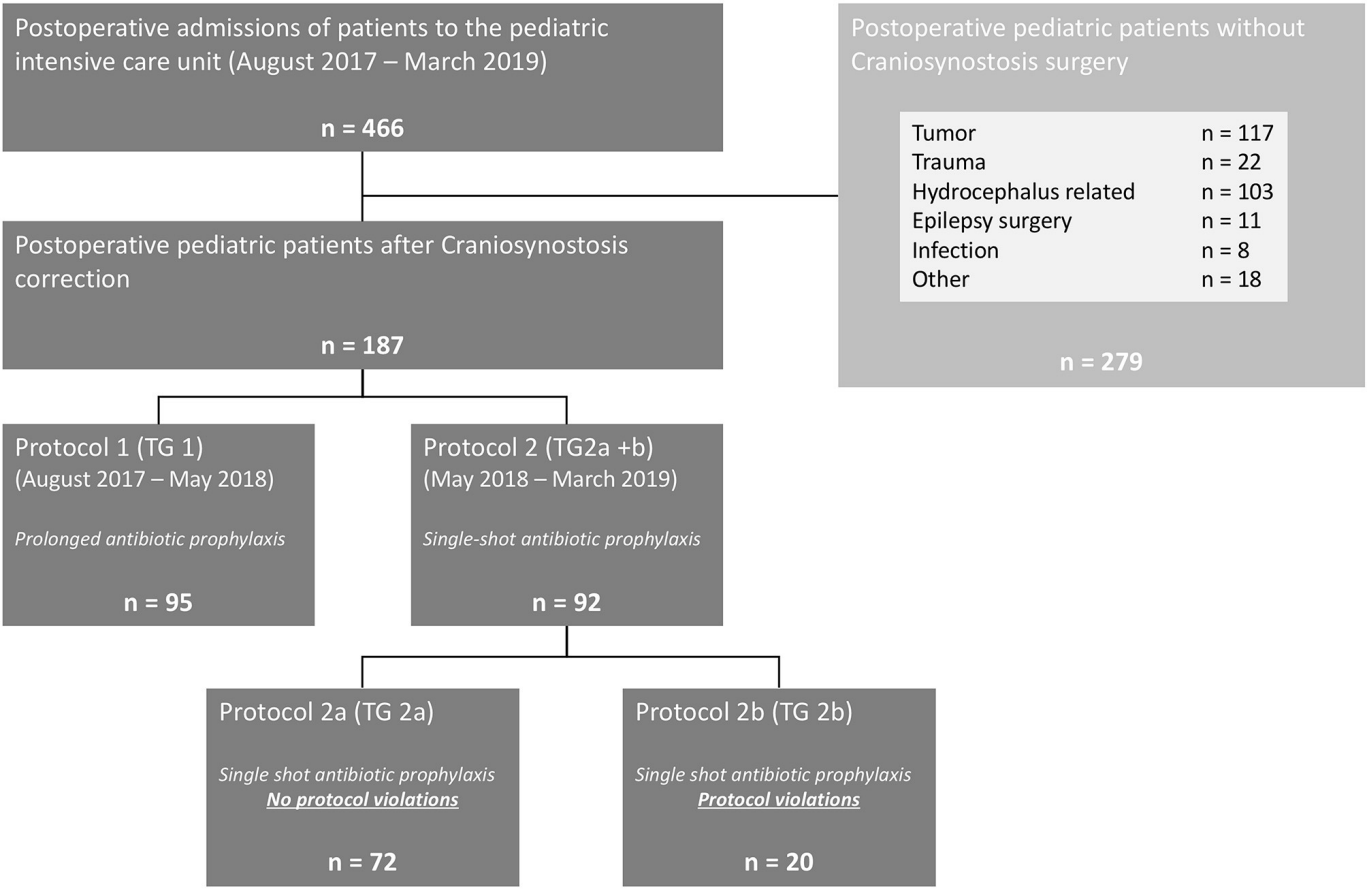
Study design and characteristics. TG, treatment group.

In total, 187 children after craniosynostosis surgery were evaluated for this study. A detailed description of the study design is given in Figure 1. Ninety-five children were evaluated after protocol 1 as TG 1 and 72 children without violations of protocol 2 were evaluated as TG 2a—resulting in a total of 167 children treated according to protocol. Twenty children of protocol 2 had to be excluded from statistical analysis due to violations of the protocol and were followed up as TG 2b. Of those, nine children (9.8% of protocol 2) received additional antibiotics doses on the surgeon's request. The remaining 11 children received antibiotics according to protocol 1 during the first weeks after change to protocol 2 (Figure 2).

Data about the occurrence of a possible surgical site infection were available for all 187 children (100%) evaluated in this study. For 180 children (96.2%), clinical follow-up took place after a mean time of 96.4 ± 29.7 days. The parents of the remaining seven children were contacted *via* telephone. The follow-up appointment was slightly later in TG 1 (102.0 ± 28.8 days) compared with TG 2a (92.1 ± 31.2 days) (*p* = 0.037).

Patient characteristics and surgical data are shown in Table 1. The median age of the whole patient cohort was 6 months (range 2–169 months), only four children were older than 2 years. The mean hospital duration was 5.1 ± 1.4, and the mean duration in ICU was 1.2 ± 0.8 days. Patients in TG 1 stayed longer in the hospital (*p* = 0.026) but shorter on the ICU (*p* = 0.010) in comparison with patients in TG 2a. The most common diagnosis was sagittal craniosynostosis with 52.4% followed by metopic suture synostosis with 24.1% of all included cases. Patients with an underlying genetic predisposition were a minority (7.5%), with Crouzon syndrome being the most common among these (1.6%). There were no significant differences of baseline characteristics between TG 1 and TG 2a. No mortality was observed for the whole group; 87.4% of the patients in TG 1 were operated on with an open technique in comparison with 76.4% in TG 2a (*p* = 0.064). Drainage volume after an open procedure was higher in TG 2a (TG 1: 120 ml (90–170) vs. TG 2a: 150 ml (103–220); *p* = 0.016).

**Table 1:** Baseline characteristics of all included cases.

	**Protocol 1 TG1**	**Protocol 2**			**Total**	***P*-value**
		**Total**	**TG 2a**	**TG 2b**		**TG 1 vs. TG 2a**
Study period (months)	9	10	10	10	19	n.a.
Number of patients	95	92	72	20	187	n.a.
Age (months)^*^	6.0 (5.0–10.0)	6.0 (3.0–11.0)	7.5 (4.3–11.0)	5.0 (3.0–11.8)	6.0 (3.0–11.8)	0.982
Body weight (kg)^*^	8.2 (7.0–9.0)	8.00 (6.9–9.5)	8.3 (6.9–9.5)	7.1 (6.5–9.1)	8.1 (7.0–9.3)	0.661
Body height (cm)^*^	70 (67–74)	70 (65–76)	71 (65–76)	67 (63–76)	70 (66–75)	0.944
**Gender**						
Female^‡^	31 (32.6%)	22 (23.9%)	20 (27.8%)	2 (10.0%)	53 (28.3%)	0.503
Male^‡^	64 (67.4%)	70 (76.1%)	52 (72.2%)	18 (90.0%)	134 (71.7%)	0.503
Hospital duration (*d*)^#^	5.3 ± 1.7	4.9 ± 1.1	5.1 ± 1.2	4.8 ± 1.0	5.1 ± 1.4	0.026
ICU duration (*d*)^#^	1.5 ± 0.9	1.0 ± 0.5	1.1 ± 0.4	1.2 ± 0.4	1.2 ± 0.8	0.010
**Diagnosis**						
Sagittal craniosynostosis^‡^	53 (55.8%)	45 (48.9%)	36 (50.0%)	9 (45.0%)	98 (52.4%)	0.461
Metopic craniosynostosis^‡^	19 (20.0%)	26 (28.3%)	21 (29.2%)	5 (25.0%)	45 (24.1%)	0.171
Coronal craniosynostosis^‡^	9 (9.5%)	12 (13.0%)	10 (13.9%)	2 (10.0%)	21 (11.2%)	0.377
Lambdoid craniosynostosis^‡^	1 (1.1%)	1 (1.1%)	1 (1.4%)	0 (0.0%)	2 (1.1%)	0.844
Bisutural craniosynostosis^‡^	11 (11.5%)	6 (6.5%)	4 (5.6%)	2 (10.0%)	17 (9.1%)	0.180
Trisutural craniosynostosis^‡^	1 (1.1%)	1 (1.1%)	0 (0.0%)	1 (5.0%)	2 (1.1%)	0.386
Pansynostosis^‡^	1 (1.1%)	1 (1.1%)	0 (0.0%)	1 (5.0%)	2 (1.1%)	0.386
**Predisposition**						
Nonsyndromic^‡^	88 (92.6%)	85 (92.4%)	68 (94.4%)	17 (85.0%)	173 (92.5%)	0.642
Crouzon syndrome^‡^	2 (2.1%)	1 (1.1%)	0 (0.0%)	1 (5.0%)	3 (1.6%)	0.218
Pfeiffer syndrome^‡^	0 (0.0%)	2 (2.2%)	0 (0.0%)	2 (10.0%)	2 (1.1%)	n.a.
Apert syndrome^‡^	0 (0.0%)	1 (1.1%)	1 (1.4%)	0 (0.0%)	1 (0.5%)	0.252
Muenke syndrome^‡^	0 (0.0%)	1 (1.1%)	1 (1.4%)	0 (0.0%)	1 (0.5%)	0.252
Craniofrontonasal dysplasia^‡^	1 (1.1%)	2 (2.2%)	2 (2.8%)	0 (0.0%)	3 (1.6%)	0.409
Other^‡^	4 (4.2%)	0 (0.0%)	0 (0.0%)	0 (0.0%)	4 (2.1%)	0.079
**Surgical data**						
Open surgery^‡^	83 (87.4%)	67 (72.8%)	55 (76.4%)	12 (60.0%)	150 (80.2%)	0.064
Surgery duration (min)^*^	89 (57–158)	139 (62–163)	143 (60–163)	137 (115–166)	118 (60–160)	0.534
Drain placement^‡^	83 (100%)	67 (100%)	55 (100%)	12 (100%)	150 (100%)	n.a.
Drainage volume (ml)^*^	120 (90–170)	150 (110–220)	150 (103–220)	170 (150–210)	140 (90–200)	0.016
Drainage duration (days)^#^	1.5 (±0.5)	1.6 (±0.5)	1.6 (±0.5)	1.7 (±0.5)	1.5 (±0.5)	0.811
Endoscopic surgery^‡^	12 (12.6%)	25 (27.2%)	17 (23.6%)	8 (40.0%)	37 (19.8%)	0.064
Surgery duration (min)^*^	60 (47–70)	52 (48–58)	52 (41–59)	50 (48–58)	52 (47–62)	0.057
**Transfusion**						
Erythrocyts^‡^ (pRBC)						
Intraoperative	35 (36.8%)	33 (35.9%)	26 (36.1%)	7 (35.0%)	68 (36.4%)	0.923
Postoperative	23 (24.2%)	19 (20.7%)	13 (18.1%)	6 (30.0%)	42 (22.5%)	0.341
**Fresh frozen plasma** ^**‡**^						
Intraoperative	31 (32.6%)	34 (37.0)	26 (36.1%)	8 (40.0%)	65 (34.8%)	0.641
Postoperative	0 (0.0%)	0 (0.0%)	0 (0.0%)	0 (0.0%)	0 (0.0%)	n.a.
**Catecholamine requirement** ^**‡**^						
Intraoperative	39 (41.1%)	59 (64.1%)	45 (62.5%)	14 (70.0%)	98 (52.4%)	0.006
Postoperative	1 (1.1%)	1 (1.1%)	1 (1.4%)	0 (0.0%)	2 (1.1%)	0.844

*Protocol 1, prolonged perioperative antibiotic treatment; Protocol 2, single-shot antibiotic treatment. TG 2a, treatment according to protocol; TG 2b, deviations from the antibiotic treatment protocol; pRBC, packed red blood cells*.

* *Variables are reported as median with interquartile range*.

# * Variables are reported as mean ± standard deviation*.

‡ *Variables are reported in the number of patients and percent of patients. n/a, not applicable*.

### Antibiotic Dosing and Adherence to Dosing Recommendations

All TG 1 children received cefuroxime with a mean first dose of 50 mg/kg body weight and 33 mg/kg body weight for every further dose. Most of the patients received four doses of cefuroxime. All TG 2a children received only one intraoperative dose of cefuroxime (50 mg/kg body weight).

### Microbiological Colonialization of Wound Drains and Wound Infection Rate

In all cases of open techniques (100%), a total of 150 wound drains were placed (Table 2). Generally, no drain was placed after an endoscopic procedure. Of the placed drains, 106 (70.7%) were analyzed either with conventional microbiological culture techniques or cultures after sonication. Of all sonicated drains (TG1: 3/63; TG2a: 4/38; TG2b: 1/5), 7.5% showed a positive culture result, *Propionibacterium acnes* being the most common pathogen (62.5%). Conventional microbiological techniques of the wound drains were performed in six patients yielding one positive culture. There was no significant difference in the drain colonialization rate between both groups neither after sonication (*p* = 0.269) nor conventional microbiological diagnostics (*p* = 0.439). In 104 patients in total (69.3%), skin swabs of the drainage entry point were performed. All cultures were negative for pathogens.

**Table 2:** Baseline infection characteristics of all included cases.

	**Protocol 1 (treatment group 1)**	**Protocol 2**		**Total**	***P*-value (treatment group 1 vs. 2a)**
		**Treatment group 2a**	**Treatment group 2b**		
Number of patients	95	72	20	187	
Open surgery	83 (87.4%)	55 (76.4%)	12 (60.0%)	150 (0.2%)	
Drain placed	83 (100%)	55 (100%)	12 (100%)	150 (100%)	
**Sonication results**					
Analysis performed	63 (75.9%)	38 (69.1%)	5 (41.7%)	106 (70.7%)	
Negative result	60 (95.2%)	34 (89.5%)	4 (80.0%)	98 (92.5%)	0.269
Positive result	3 (4.8%)	4 (10.5%)	1 (20.0%)	8 (7.5%)	
Propionibacterium acnes^‡^	1 (33.3%)	3 (75.0%)	1 (100.0%)	5 (62.5%)	
*Staphylococcus epidermidis* ^‡^	1 (33.3%)	0 (0.0%)	0 (0.0%)	1 (12.5%)	
*Staphylococcus saprophyticus* ^‡^	1 (33.3%)	0 (0.0%)	0 (0.0%)	1 (12.5%)	
*Micrococcus luteus* ^‡^	0 (0.0%)	1 (25.0%)	0 (0.0%)	1 (12.5%)	
**Conventional microbiological results**					
Analysis performed	2 (2.4%)	4 (7.3%)	2 (16.7%)	8 (5.3%)	
Negative result	2 (100.0%)	3 (75.0%)	2 (100.0%)	7 (87.5%)	0.439
Positive result	0 (0.0%)	1 (25.0%)	0 (0.0%)	1 (12.5%)	
*Staphylococcus epidermidis*	0 (0.0%)	1 (100%)	0 (0.0%)	1 (100%)	
**Skin swab results**					
Analysis performed	58 (69.9%)	40 (72.7%)	6 (50.0%)	104 (69.3%)	
Negative result	58 (100.0%)	40 (100.0%)	6 (100.0%)	104 (100.0%)	n/a
Positive result	0 (0.0%)	0 (0.0%)	0 (0.0%)	0 (0.0%)	
**Wound infection**					
Positive result	1 (1.1%)	0 (0.0%)	0 (0.0%)	1 (0.5%)	0.383

*n/a, not applicable*.

‡ *Variables are reported in the number of patients and percent of patients*.

Protocol 1, prolonged perioperative antibiotic treatment; protocol 2, single-shot antibiotic treatment; Treatment group 2a, treatment according to protocol; Treatment group 2b, violations from the antibiotic treatment protocol 2. Statistical comparisons were calculated between treatment group 1 and treatment group 2a. Data are reported as absolute number of patients (and percentage).

During the 3-month follow-up, only one surgical site infection was documented, which corresponds with an overall wound infection rate of 0.5%. The wound infection occurred 108 days postoperatively with a localized superficial wound dehiscence (*Staphylococcus aureus*) necessitating local surgical revision. Primarily, this child had undergone open correction for a combined metopic and unilateral coronal suture synostosis and was treated according to protocol 1 (seven doses of antibiotic prophylaxis). The initial sonication and wound swab analyses were negative. There was no significant difference in wound infection rates between TG 1 (1.1%; 1/95) and TG 2a (0.0%; 0/72) (*p* = 0.383).

### Perioperative Development of Laboratory Markers

**Figure 3:**
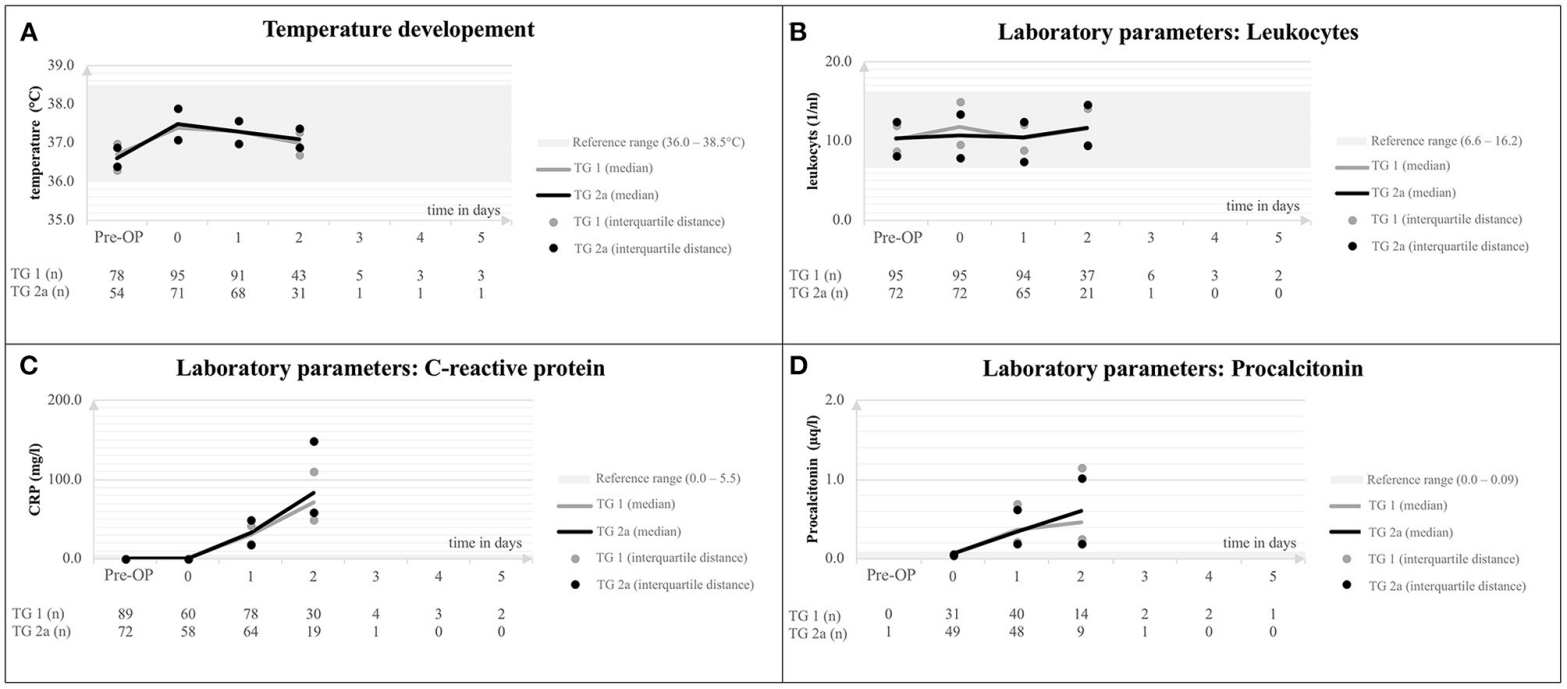
Body temperature **(A)**, leukocyte counts **(B)**, C-reactive protein **(C)**, and procalcitonin **(D)** after craniosynostosis surgery compared between TG 1 and 2a. TG, treatment group.

After surgery, children showed a minor increase of body temperature which normalized within 48 h after surgery in most children. Within 24 h, there was a marked increase of CrP in both treatment groups to a median of 70.9 mg/l (range 49.4–110.3) for TG 1 and a median of 82.7 mg/l (59.2–149.6) for TG 2a (*p* = 0.091). Similarly, patients showed an increase of PCT after surgery to a median PCT of 46 μg/l (0.25–1.15) in TG 1 and a median PCT of 0.61 μg/l (0.19–1.02) in TG 2a (*p* = 0.442) 24 h after surgery (Figure 3). None of the patients had clinical signs of acute local infection.

## Discussion

In this study, 187 children were enrolled after craniosynostosis surgery, investigating the role of two different PAP protocols. Although the duration of PAP was reduced from a mean of four doses to an intraoperative single shot of cefuroxime only, there were no significant differences neither in the rate of wound infections nor in the rate of microbial colonization of the skin or wound drains during the follow-up.

Postoperative surgical site infections are a major problem for patients as well as the healthcare system (6). It has been shown that the occurrence of a wound infection increases the average length of the hospital duration by about 6 days. Postoperative wound infections are the most common reason for a readmission in the field of pediatric craniofacial surgery (7). The reported postoperative wound infection rate after the correction of a craniosynostosis ranges from 0.2 to 15.5% in the current literature (8, 9). In general, for the majority of craniosynostosis patients—those with single-suture craniosynostosis—surgical correction is electively performed in an otherwise healthy child. In such a setting, no perceptible complication rate including surgical site infections can be accepted and the administration of PAP aims to prevent its occurrence. The wound infection rate in our cohort (0.5%) was at the lower range of the previously published results (8, 9).

There are numerous studies evaluating risk factors that predispose for postoperative wound infection. Recurrent surgical corrections have higher infection rates as well as patients with an underlying genetic syndrome (10). Socioeconomic factors predispose toward a delayed primary surgery, which again causes an increase of the 30-day readmission rate and the risk for a surgical site infection (11). PAP is known to have an influence on wound infection rate as well, but distinct recommendations regarding the duration of PAP in craniofacial surgery are missing in the most recently published guideline (12). A recent systematic review of 39 studies with a total of 4,336 patients after head and neck surgery sought to elicit the optimal prophylactic antibiotic regimen for surgical site infections. The authors demonstrated that a prophylactic antibiotic treatment longer than 48 h after surgery did not further reduce the wound infection rate (13). Studies investigating the prophylactic administration of antibiotics in orthopedic surgery only proved a positive effect for a treatment duration of up to 24 h after surgery (14). With the increasing number of multiresistant pathogens worldwide, efforts are made to limit the inadequate use of antibiotics (15), so duration, type, and dosage of antibiotics used have come under the scrutiny of clinicians. Antibiotic stewardship programs attempt to collect all existing evidence and to shorten the prophylactic antibiotic exposure (16). The implementation of pediatric antimicrobial stewardship programs allowed a reduction of targeted and empiric antibiotic use, a decrease in healthcare cost, and fewer antimicrobial resistances (17). In the light of those findings, the duration of antibiotic prophylaxis after all craniosynostosis corrections was shortened after May 2018. It was our hypothesis that a reduction of the perioperative antibiotic prophylaxis to a minimum of a single preoperative dose would not cause an increased surgical site infection rate. As surrogate parameters for a possible early infection after surgery body temperature, leukocyte count, CRP and the procalcitonin level of both groups did not show any differences between the two treatment protocols. Contrary to the work of Kalantar et al. and Mekitarian Filho et al., we did not experience a postoperatively increased body temperature within the first 2 days after craniosynostosis correction as a clinical sign of an impending infection (18, 19). We agree with the interpretation of Maday et al. and With et al. that postoperatively increased body temperature rather reflects physiological systemic reaction upon surgical trauma than beginning infection; an increase of body temperature postsurgery should not automatically trigger anti-infective therapy (20, 21). This argument is supported by the fact that while most of our patients developed this temporary inflammatory reaction only one patient developed a manifest surgical site infection. It is known that the individual perception of a risk for postoperative site infection may affect the prescribing habits. Especially, the use of postoperative surgical drains is known to correlate strongly with the duration of antibiotic prophylaxis (22). In the past, prolonged drain placement was associated with bacterial colonization and wound infections in general surgery (23). The rate of positive cultures after sonication of the evaluated drains was higher in group 2a (10.5%) than in group 1 (4.8%) but without reaching significance (*p* = 0.269) and without resulting in a different rate of postoperative surgical site infection (0 vs. 1.1%, *p* = 0.383). In the light of this most important finding and with an overall rate of only 7.5% positive microbiological results of the evaluated drains in total, we conclude that existence of a surgical drain in place does not justify prolonged PAP regimen beyond a single-shot antibiotic dosage after craniosynostosis correction. This statement is supported by the 100% rate of negative microbiological cultures of the skin swabs from the drainage site in examined children. Of note, cefuroxime would not be recommended in the treatment of infections induced by any of those bacteria which we have found in the wound drains of our patients. Our study was neither designed nor statistically powered for an evaluation regarding the choice of perioperative prophylaxis, so we cannot derive any suggestions hereon based on our microbiological findings. The decision about which antibiotic drug is the most appropriate for perioperative prophylaxis would need to be investigated in a prospective trial specifically adressing this important research question.

The presented study has the following limitations. The prospectively collected data for the introduced protocol 2 were compared with a historical cohort of protocol 1, for which the data were only retrospectively collected. After the introduction of protocol 2, the operating surgeons decided to prolong PAP in nine patients due to an assumed elevated risk of infection, which resulted in protocol violations and may result in a possible selection bias. In addition, the observed low rate of infections (0.5%), would require very high numbers of study participants to substantially increase the power of the study, which is hardly feasible for a single center within a reasonable timeframe.

However, the findings of the presented study may serve to project a future, multicentered randomized trial set-up.

## Conclusion

To the best of our knowledge, this is the first prospective study comparing two different PAP protocols in pediatric patients after craniosynostosis surgery. The reduction of PAP to a preoperative single-shot administration of cefuroxime was not inferior to an extended PAP regimen regarding the rate of surgical site infections or microbial colonization of wound drains. Based on our findings, a reduction of PAP to a single-shot regimen seems adequate and safe for distinct surgical procedures, allowing a reduction of antibiotic use in the context of antibiotic stewardship.

## Data Availability Statement

The raw data supporting the conclusions of this article will be made available by the authors, without undue reservation.

## Ethics Statement

The studies involving human participants were reviewed and approved by Ethikkommission der Charité-Universitätsmedizin Berlin (EA2/029/18). Written informed consent to participate in this study was provided by the participants' legal guardian/next of kin.

## Author Contributions

JH and TF: study projection, data acquisition and interpretation, and drafting manuscript. JL: data acquisition and interpretation, drafting manuscript, and statistical evaluation. FS: study projection, data acquisition and interpretation, and supervision. AS: data acquisition and interpretation and review of manuscript. AG: study projection, data acquisition and interpretation, and review of manuscript. HB and U-WT: study projection, supervision, and review of manuscript. MS: study projection, data acquisition and interpretation, drafting manuscript, and review of manuscript. All authors contributed to the article and approved the submitted version.

## Conflict of Interest

The authors declare that the research was conducted in the absence of any commercial or financial relationships that could be construed as a potential conflict of interest.

## Publisher's Note

All claims expressed in this article are solely those of the authors and do not necessarily represent those of their affiliated organizations, or those of the publisher, the editors and the reviewers. Any product that may be evaluated in this article, or claim that may be made by its manufacturer, is not guaranteed or endorsed by the publisher.
